# From Global-to-Local? Uncovering the Temporal Dynamics of the Composite Face Illusion Using Distributional Analyses

**DOI:** 10.3389/fpsyg.2019.02331

**Published:** 2019-10-30

**Authors:** Daniel Fitousi

**Affiliations:** Department of Behavioral Science, Ariel University, Ariel, Israel

**Keywords:** composite faces, delta plots, global-to-local, feature-based processing, distributional analyses, reaction time, holistic processing

## Abstract

It is widely believed that faces are processed holistically such that their facial features or parts are represented as global wholes rather than independent entities. But how does their holistic representation evolve in time? According to the *global-to-local* hypothesis, the initial representation of faces is holistic and coarse at the outset but is becoming progressively detailed and analytic. The current study set to test this *global-to-local* hypothesis by applying fine-grained methods of response time analyses to the *composite face illusion* – a traditional marker of holistic face processing. The analyses included the *delta plots* and *conditional accuracy functions.* These tools move beyond the mean RT and accuracy to provide detailed analysis of the temporal dynamics of the composite face effect. The methodologies converged on the conclusion that the composite effect is minimal for fast RTs but becomes progressively larger as RT gets slower. This pattern is inconsistent with a global-to-local dynamics. The implications of these results to the study of face perception are discussed.

## Introduction

Faces convey a great deal of information regarding a host of social and emotional aspects (e.g., expression, gender). Rapid and accurate perception of faces is therefore essential for survival. A fundamental goal of psychologists (Tanaka and Farah, 1993) and neuroscientists alike (Kanwisher et al., 1997; Tsao and Livingstone, 2008) is to uncover the basic mechanisms that govern face perception. According to the dominant holistic approach, faces are processed and perceived as unitary wholes rather than parts or features (Farah et al., 1998; Maurer et al., 2002). Proponents of this view argue that the facial features or parts are not perceived as independent entities, but rather as an interconnected Gestalt. Despite the vast research on this topic, the mechanisms that support holistic processing are not well-understood (Tanaka and Farah, 1993; Carey and Diamond, 1994; Fitousi, 2015, 2016a).

One important question that stands out in the study of faces concerns the temporal dynamics of the underlying representation. Is a face represented initially as a collection of independent features which are later integrated into a holistic representation? Or, alternatively, is a face represented holistically from the outset? And if so, does it remain holistic to the same degree over time? According to the *global-to-local* hypothesis (Sergent, 1986) – held by many advocates of holistic perception (Jacques and Rossion, 2009) – the representation of a face is coarse and holistic at the early stages of processing, but then becomes progressively detailed and amenable to analytic perception.

The evidence for this *global-to-local* hypothesis is rather mixed and indirect (Sergent, 1986; Searcy and Bartlett, 1996; Goffaux et al., 2005; Goffaux and Rossion, 2006; Jacques et al., 2007; Jacques and Rossion, 2009; Richler et al., 2009; Meinhardt-Injac et al., 2010, 2011). The current study sought to shed light on the *global-to-local hypothesis* through the application of fine-grained analyses of response times distributions (Balota and Yap, 2011) to one of the most prominent phenomenon of holistic face processing – the *composite face illusion* (Young et al., 1987). To date, testing with the composite faces has been confined to mean RTs or mean accuracy rates (Rossion, 2013), while the important information lurking in the RT distributions has been overlooked (but see Fitousi, 2015). This is quite surprising given the extensive applications of distributional analyses in other fields of cognitive research (Ratcliff, 1978), as well as the widespread interest in face perception (Bruce and Young, 2013). The current work sought to fill in at least part of this lacuna.

### Face Recognition Effects

There are three main experimental effects that have been routinely used to support the notion of holistic or global processing: (a) the *inversion effect* (Yin, 1969), in which recognition of inverted faces is hampered relative to upright faces, (b) the *part-whole* effect (Tanaka and Farah, 1993), in which the recognition of facial parts is improved when presented in the context of the entire face rather than isolated, and (c) the *composite face effect* (Young et al., 1987), in which recognition of the top part of a composite face is hampered when the bottom part belongs to a different face. It is reasonable to ask how these empirical phenomena develop in time. Uncovering the temporal dynamics of these standard markers of allegedly holistic processing can greatly inform face perception theories. Do these effects emerge at once and then decrease with time? Or, alternatively, absent at the outset, but gain presence and influence with time?

The current study set to answer these questions with respect to one of the three phenomena: the *composite face effect* (Young et al., 1987). The present effort applies a set of fine-grained distributional analyses on the entire response latencies in the composite face task. The tests consist of the *cumulative distribution functions* (CDFs, Townsend and Ashby, 1983), *delta plots* (Pratte et al., 2010), and *conditional accuracy functions* (CAFs, Ridderinkhof, 2002a). The aim is to uncover the time course of the composite face effect. These tools have been routinely applied to the Stroop, flanker and Simon effects producing important theoretical insights (Pratte et al., 2010; Balota and Yap, 2011). A similar application of these tools to the composite face illusion will allow us to go beyond the mean RTs and to exploit the important information lurking in the entire distributions. We will also be able to test the *global-to-local* hypothesis (Sergent, 1986) in a powerful way, and compare the temporal dynamics of the composite face illusion to that of other attentional effects (e.g., Stroop) to which it has been related (see Rossion, 2013). The structure of the remaining sections is as follows: (a) a short overview of the composite face effect, (b) an outline of the evidence for and against global-to-local processing of faces (c), an introduction of the distributional tools employed in the current study, and (d) an outline of the tested predictions.

### The Composite-Face Effect

**FIGURE 1:**
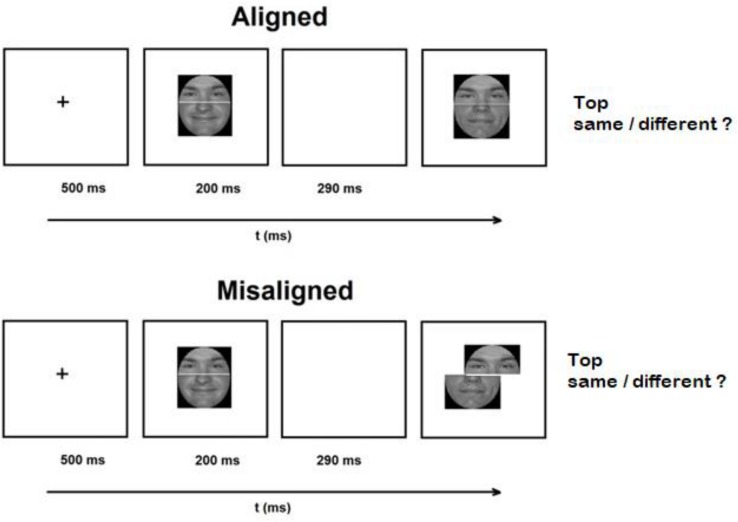
An illustration of an experimental trial with unfamiliar aligned and misaligned composite faces.

In a seminal paper by Young et al. (1987), top and bottom halves from two famous people were presented. Naming the top half of the face was slowed down when the parts were aligned compared to when the parts were misaligned. The effect was dubbed an “illusion” because in the aligned condition, the two famous faces’ halves formed a new unfamiliar face. Hole (1994) extended this effect to unfamiliar faces, using a matching task in which participants were presented with a study face and then a test face. The participants’ task was to decide whether the top half of the test face is ‘same’ or ‘different’ from the top half of the study face (see Figure 1). In this task, it is often more difficult to judge whether the top halves of two faces are same or different when the two halves are aligned with different bottom halves than when they are misaligned. The composite face phenomenon is arguably one of the most powerful pieces of evidence in favor of holistic face processing (Hole, 1994; Weston and Perfect, 2005; Michel et al., 2006; Richler et al., 2008; Curby et al., 2013). This illusion and the tasks that have been administrated to measure it have gained the status of a standard in various areas of face perception such as: development (Mondloch et al., 2007; Cassia et al., 2009), populations with special impairment (Schwartz et al., 2002; Avidan et al., 2011), social cognition (Bukach et al., 2012), and modeling of face recognition (Dailey and Cottrell, 1999; Fitousi, 2013).

**FIGURE 2:**
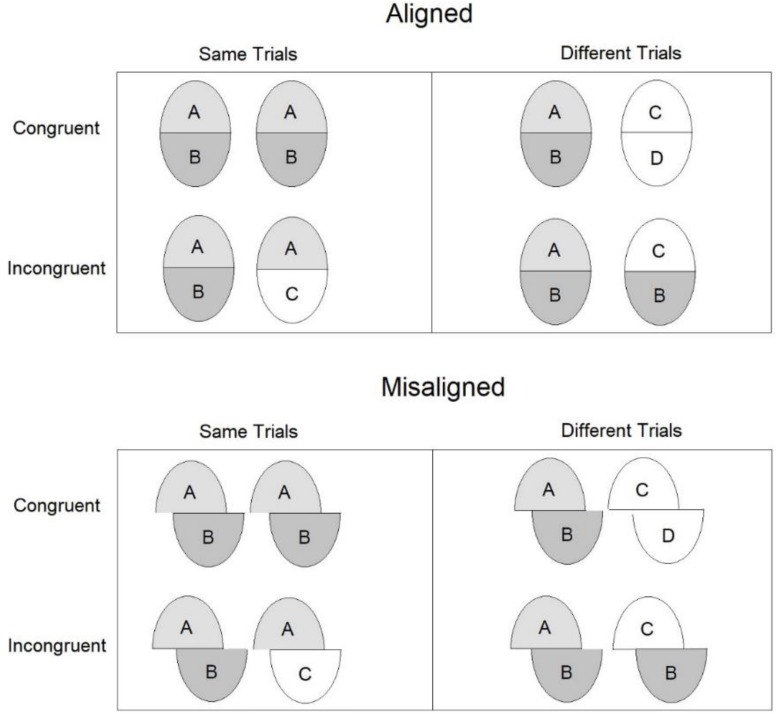
Overview of the design of the sequential matching task. Each pair of faces designates a study and test pair. The complete design measure is based on all pairs, whereas the partial design measure is based only on the trials in the ‘same – incongruent’ conditions for aligned and misaligned composites.

Research on composite faces has deployed two major and allegedly incompatible measures of holistic processing in the matching paradigm. Both can be derived from the same experiment (see Figure 2). The first is based on the so called “*partial design”* (Rossion, 2013). It is computed as a difference in performance between aligned and misaligned conditions only for trials in which the relevant half (e.g., top) is ‘same’ and the irrelevant half (e.g., bottom) is ‘different.’ The measure captures the persisting impression that the relevant (top) halves in two composites are not the same (although they are) when the relevant (top) halves are composed with different irrelevant (bottom) halves. Thus, it is more difficult to respond ‘same’ in aligned condition than in a misaligned condition. This is because in the former configuration perception of the relevant half is dependent on the irrelevant part. This effect likely reflects a template or Gestalt-like representation, where face parts and features are encoded as a single unit (Rossion, 2013).

The second measure is based on the so called *“complete design”* version (Richler and Gauthier, 2014). It is computed as a Congruency × Alignment interaction term. This term captures the idea that for aligned composite faces performance is better with congruent (i.e., both top and bottom parts are ‘same’ or both are ‘different’) than with incongruent (i.e., top is ‘same’ and bottom is ‘different’ or vice versa) faces. The congruency effect is decreased or completely abolished with misaligned faces. This pattern has been interpreted as reflecting the operation of a selective attention mechanism (Chua et al., 2014). Selective attention to the irrelevant half fails because observers have learned to associate aligned face parts due to extensive experience with faces. Signal detection (Green and Swets, 1966) indices of d’ and c have been often used to compute the effect in the complete design (Richler et al., 2009) to dissociate discriminability from response bias.

The debate between proponents of the two measures is still unsettled. Rossion (2013) argued that the complete design measure is untannable because the congruity effect reflects a response conflict akin to other attentional conflict measures such as the Stroop (1935) and flanker (Eriksen and Eriksen, 1974). Richler and Gauthier (2014), on the other hand, have argued that the partial design confounds response bias and congruency because in the partial design the irrelevant part is always “different,” rendering all relevant “same” trials incongruent and all relevant “different” trials congruent. In addition, in the language of SDT, all ‘same’ trials are ‘hits’ and all ‘different’ trials are ‘false alarms.’ As a result, all ‘same’ trials (hits) are incongruent, and all “different” trials (false alarms) are congruent. Hence a correct response is completely confounded with congruency.

The incongruency between the partial and complete design measures seems even more serious given the finding that the two measures exhibit insignificant low correlation (Richler and Gauthier, 2014). But do the two effects really measure different things? After all, their computations are based on the same experiment. The current study can shed light on this question by tracing their temporal dynamics. A finding showing the two measures to have utterly different time courses, would support their distinct meaning, while a finding showing similar time courses would weaken such a hypothesis. Note that the conclusions drawn by Richler and Gauthier (2014) relied on SDT measures only. To foreshadow the current results, the present study revealed comparable temporal dynamics and high to medium correlations between the two type of measures in both accuracy and RT measures.

It should be noted at this point that there are viable theoretical alternatives to holistic accounts (Fitousi, 2015). Several researchers have subjected the holistic account to strong tests against a well-defined theory-based definition of holism (Wenger and Ingvalson, 2002; Fitousi, 2016a; Cheng et al., 2018; Von Der Heide et al., 2018). Tests on entire RT distributions have been developed as part of a powerful stochastic model known as the *system factorial technology* (SFT, Townsend and Nozawa, 1995).^1^ Holistic processing in SFT entails a distinct process model called coactivation (Miller, 1982; Townsend and Nozawa, 1995). In coactive systems information from two facial features or parts should coalesce into a single channel, producing well-known effects of supercapacity and coactivation (Townsend and Nozawa, 1995; Fitousi, 2015; Fitousi and Algom, 2018, 2019) on response latencies distributions (Miller, 1982, 1986; Townsend and Wenger, 2004). When tested against this well-defined model, faces have failed to exhibit expected holistic signatures (Fitousi, 2015; Cheng et al., 2018). Consequently, Fitousi (2015, 2016a) has proposed an alternative account of the composite face effect in terms of an object-based attention effect (Duncan, 1984). According to Fitousi (2015, 2016a) the effect reflects a selective attention mechanism. In that sense the composite face effect is comparable to a Stroop effect which is akin to response conflict or facilitation (depending on context). The distributional analyses held here can shed light on this claim.

### The Global-to-Local Hypothesis

Faces provide viewers with two main sources of information – featural and configural (Bartlett and Searcy, 1993; Macho and Leder, 1998; Rakover, 2002). The former refers to the local elements that compose a face such as eyes or nose, while the latter refers to the emerging global representation. The composite face effect is arguably one of the most powerful manifestations of the holistic information, since the whole face interferes with decisions about the relevant part (e.g., top half). There is a general agreement among face researchers that observers process both featural (local) and holistic (global) information (Maurer et al., 2002). However, current approaches differ with respect to temporal order by which they are processed. According to the classic holistic approach (Tanaka and Farah, 1993; Farah et al., 1995) features are encoded initially as a Gestalt and the featural information can be extracted with much difficulty because observers need to tease them apart from the global representation (Tanaka and Farah, 1993). Sergent (1986) has proposed a “microgensis” account which outlines the temporal dynamics of global and local information. Sergent’s account relies on the discovery of visual filters for high and low frequencies in the vision system (De Valois and De Valois, 1980). Low spatial frequencies capture coarse aspects of the image whereas high spatial frequencies convey detailed and fine-grained characteristics of the image. According to Sergent (1986) holistic or global aspects of faces are conveyed by low spatial frequencies in the image, whereas featural or analytic aspects of a face are captured by high spatial frequencies in the image (see also Goffaux and Rossion, 2006). Both types of information are extracted by the visual system. However, the low-pass information is available to the system earlier than the high-pass information. This means that the recognition of a face proceeds from a coarse and holistic representation to a more detailed and analytic representation (Navon, 1977; Schyns and Oliva, 1994; Sugase et al., 1999; Jacques and Rossion, 2009).

An alternative to the global-to-local hypothesis is the *feature-based approach* (Tversky and Krantz, 1969; Diamond and Carey, 1986; Bartlett and Searcy, 1993; Rakover and Teucher, 1997; Macho and Leder, 1998; Rakover, 1998). According to this approach, the processing of features is very fast and therefore precedes the processing of global-holistic information which occurs only after the features are bound together. This approach relies on traditional theories of attention which assume that vision evolves from an automatic stage in which features are recorded in an effortless and fast mode, to a controlled stage in which object construction occurs with the effortful allocation of resources and bindings of features (Treisman and Gelade, 1980; Marr, 1982; Biederman, 1987; Fitousi, 2018). According to this approach, the processing of faces can be termed ‘local-to-global.’ The evidence for and against the global-to-local hypothesis in face perception domain is presented next.

### Evidence for and Against a Global-to-Local Dynamics

I start by reviewing the evidence supporting the global-to-local hypothesis. Building on the ideas of Sergent (1986); Goffaux and Rossion (2006) have measured the size of the composite face effect for faces that were filtered for either low or high spatial frequencies. They found disproportionately larger composite face effects for low spatial frequencies faces. Their conclusion was that holistic processing of faces is largely supported by low spatial frequencies (Goffaux et al., 2005; Goffaux and Rossion, 2006). These results can be taken as evidence for a global-to-local dynamics if one assumes that early stages of vision are governed by low spatial frequencies and later stages of vision are supported by high spatial frequencies. A more direct evidence for this notion comes from an fMRI study by Goffaux et al. (2011). They flashed masked faces for 75, 150, or 300 ms. The faces were filtered to preserve either low, medium, or high spatial frequencies. Face-preferring areas responded to coarse low spatial frequency faces at early stages of visual processing (flash duration was less than 75 ms) and decayed after that, whereas other face areas (bilateral fusiform face regions and bilateral fusiform face regions) have responded to high spatial frequency faces in later time (more than 300 ms), and their response became robust over time. These results led Goffaux et al. (2011) to argue for a coarse-to-fine strategy taken by humans in the processing of faces and objects.

Taking a different tack on the issue, Jacques and Rossion (2009) used ERP recordings in composite face task. On each trial, they presented a study and then a test composite face and measured the participants’ response to the top part. Their prediction was that if faces are processed holistically at the very early processing stages, then release from adaptation for composite faces with same top but different bottom should surface significantly on the N170 signal, which is considered to reflect early stages of processing (Bentin et al., 1996). Their logic was based on the idea that participants would perceive the two faces as two different identities and therefore should start process the new identity immediately. This is exactly what they found in the right hemisphere. Based on these results, Jacques and Rossion (2009) concluded that the perception of a face is holistic from the outset. Note however that although appealing, this study does not tell us what happens to the strength of the holistic representation with time, but only that signatures of holism show up early.

Hole (1994) manipulated the exposure durations of inverted and upright composite faces. A composite face effect was observed for brief presentation times (80 ms), but not for long (2 s) presentation times. Long exposure durations of composite faces were associated in this study with significantly slower RTs than short exposure durations. Meinhardt-Injac et al. (2010, 2011) used a part-whole task (Tanaka and Farah, 1993). In this task observers respond to a facial feature (e.g., eyes) that can appear either embedded in a face or presented in an isolated fashion. It is often found that performance is superior when facial features are presented in the context of a face. Meinhardt-Injac and colleagues found that the whole-part effect decreased as exposure duration increased. They have interpreted this result as further evidence for a global-to-local dynamics.

Evidence against the global-to-local hypothesis has been adduced by Richler et al. (2009). They varied the exposure durations of composite faces for both the study and test faces in aligned configurations. They found large holistic effects for brief presentation time, but in contrast to the global-to-local hypothesis, these effects were not attenuated in longer exposure durations.

Carbon and Leder (2005) have used “thacherised” faces in which the eyes and mouth are rotated 180° within the face. These faces often elicit an impression of grotesqueness when presented in an upright position (Thompson, 1980). However, when thacherised faces are turned upside down observers are less likely to find them grotesque, probably due to a shift from holistic to analytic mode of processing (Bartlett and Searcy, 1993; Leder and Bruce, 1998). Capitalizing on this phenomenon, Carbon and Leder (2005) conjectured that if early processing of features is beneficial for the identification of the face, then inverted thacherized faces should be processed faster than their original counterparts. They have compared performance with normal and thacherised faces both inverted, at either brief (26 ms) or long (200 ms) exposure durations. They found that at short exposure duration performance was better with inverted thacherised faces than with inverted normal faces. In contrast, in longer exposure duration, the opposite was recorded. The authors interpreted these results as suggesting that holistic (global) and featural (local) information are available at different moments in time. Local information is available at early stages, therefore at brief exposure duration a holistic representation has still not be generated and the observers based their decision on featural information, while in long exposure duration, a holistic representation has already been created, and observers used it to make their decision.

### The Present Study

As the brief review shows, previous studies do not seem to provide strong support for either the global-to-local or the local-to-global hypotheses. The nature and temporal dynamics of the (holistic?) mechanism that generates the composite face effect are still not well-understood. Previous research has been mainly preoccupied with central tendencies measures such as mean accuracy or mean RT. But mean RTs can conceal important trends that are lurking in the data (Balota and Yap, 2011; Fitousi and Wenger, 2011). Moreover, the means cannot tell us much about the dynamical aspects of processing. Hence, analyzing of the composite face effect at the level of the distributions rather than the mean can be highly valuable (Luce, 1986). First, it will afford a fine-grained analysis of the composite face effect, revealing how it is altered by processing time. Second, the emerging patterns at the distributional level can be used to test entire classes of models or hypotheses. The following sections introduce the distributional tools that will be deployed in the current study. These sections will be followed by the qualitative predictions for the global-to-local and local-to-global hypotheses.

### Cumulative Distributions Functions (CDFs)

**FIGURE 3:**
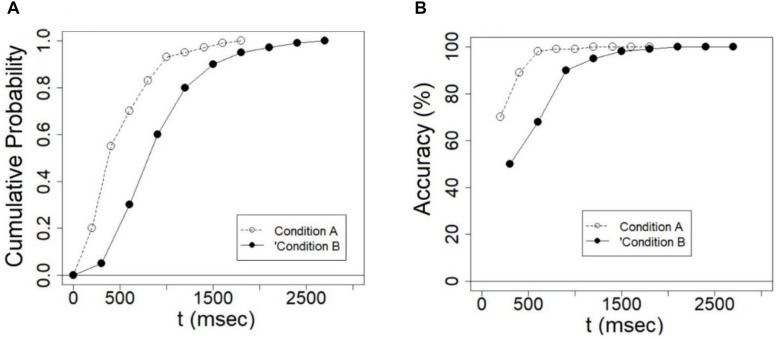
**(A)** Examples of *cumulative probability functions* for a fast condition A and a slow condition B. The former reaches its asymptote earlier than the latter. The difference between the functions at a given time indicates the size of the effect at that time. **(B)** Examples of *conditional accuracy functions* (CAFs) for fast and slow conditions. Differences in accuracy at a given time indicate the size of the effect in terms of accuracy at that time.

The *cumulative distribution function F(t)* “gives the probability that completion [of a task] occurs at a time less than or equal to *t*” (Townsend and Ashby, 1983, p. 24). The CDF is a valuable tool in modeling stochastic processes because it enables researchers to study the time course of response by treating it as a probabilistic process (Miller, 1982; Luce, 1986; Fitousi and Wenger, 2013; Algom and Fitousi, 2016; Fitousi and Algom, 2018). To plot the CDF of a given condition, the RTs are ordered from the fastest to the slowest. Then, the RT values that stand in the 10, 20, ……90% percentile are extracted. Next, the RT percentile values are plotted against their cumulative probability values. Figure 3A illustrates an example in which CDFs from two conditions (e.g., congruent vs. incongruent) are plotted. The abscissa represents time *t* and the ordinate represents the probability of completion at that time [P (**T** ≤ t)]). The magnitude of an effect can be inferred visually by either comparing the completion times of the two conditions in a given percentile, or alternatively, by comparing the cumulative probabilities at a given time *t*. If these differences remain constant across completion times in the former case, or across percentiles in the latter case, then the magnitude of the effect remains the same across time. If, on the other hand, these differences increase or decrease with time or percentiles, then the effect grows or expires with time, respectively (Balota and Yap, 2011). A global-to-local dynamics should be manifested as a decreasing effect in the CDFs. A local-to-global dynamics should exhibit an increasing effect in the CDFs.

### Conditional Accuracy Functions (CAFs)

*Conditional accuracy functions* plots the accuracy of responding as a function of response time (Luce, 1986; Ridderinkhof, 2002a). To plot the CAF, response times are ordered from the fastest to the slowest. Then, both the pertinent RT and accuracy values are computed for each of the 10–90% percentiles. Next, these values are plotted one against the other. It is often found that accuracy is near chance for fast RTs and increases as RTs get slower. This asymptotic performance might be the result of fast guess in fast responses that are replaced by more informed responses. However, there are cases in which accuracy does not increase or reach an asymptotic level. Figure 3B illustrates a fast and slow conditions in which the former reaches the asymptote faster than the latter. CAF plots can shed light on the dynamic relations between accuracy and speed and on the magnitude of the effect in terms of accuracy as a function of time. If indeed composite faces are processed according to a global-to-local dynamics, then the size of the composite face effect in terms of accuracy should decrease with time. This is because as the holistic representation becomes weaker, the interference from the irrelevant face part decreases and the composite effect should get smaller. The opposite should be found if composite faces are processed according to a local-to-global dynamics.

### Delta Plots

**FIGURE 4:**
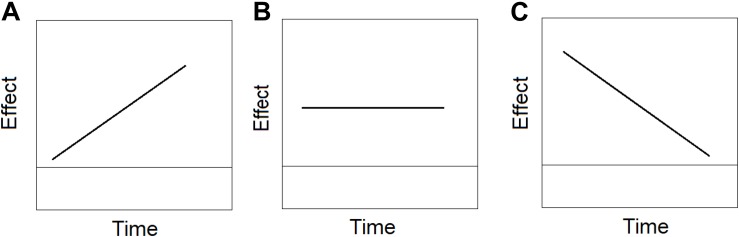
Examples of **(A)** positive slope delta plot in which the effect increases with time, **(B)** zero slope delta plot in which the effect has the same magnitude across time, and **(C)** negative slope delta plot, in which the effect decreases with time.

Delta plots (DPs) have been first used by De Jong et al. (1994) to probe the size of the Simon effect (e.g., mean RT incongruent – mean RT congruent) as a function of time. To produce a delta plot, the difference between two conditions in mean RTs (e.g., mean RT_1_- mean RT_2_) is computed in each percentile (10, 20 …90%). These differences are plotted against the mean of the two mean RTs [(mean RT_1_ + mean RT_2_)/2]. Delta plots can be positive negative or zero, as illustrated in Figure 4 (Ridderinkhof, 1997; Zhang and Kornblum, 1997; Ridderinkhof et al., 2005; Speckman et al., 2008; Pratte et al., 2010). A positive delta plot implies that the effect is increasing with time. A negative delta plot implies that the effect is decreasing with time. And a zero-slope delta plot entails that the effect remains constant in size across time (see Figure 4) (Pratte et al., 2010). The slope of the delta plot can provide important information. For example, it has been found that delta plots for the Stroop effect (Stroop, 1935) have a positive slope, whereas delta plots for the Simon effect (Simon and Rudell, 1967) have a negative slope (but see Zhang and Kornblum, 1997; Fitousi, 2016b), leading researchers (Pratte et al., 2010) to argue that albeit their similarity, the two phenomena are generated by different processes. The reason is that the same cognitive architecture cannot produce both negative and positive (or zero) slope (Schwarz and Miller, 2012). And therefore, the shape of the delta plot can assist in disentangling process models for phenomena that mimic each other at surface level.

Moreover, the slope of the delta plot (positive, negative, zero) can shed light on the underlying processes, supporting or refuting entire classes of processing models (Speckman et al., 2008; Schwarz and Miller, 2012). For example, all positive and increasing delta plots are compatible with either drift rate or decision bound changes in the diffusion model^2^ (Ratcliff, 1978; Rouder, 1996). A negative slope of the delta plot refutes a simple diffusion model (Rouder, 1996; Pratte et al., 2010; Ulrich et al., 2015), because it necessitates complex modifications of several parameters in the model. Hence, delta plots can help researchers advance models of conflict phenomenon.

Given the diagnostic power of the delta plot, it would be valuable to ask whether the delta plots of the composite face effect in the partial and complete designs are comparable to those recorded in the Stroop or Simon effects. For example, based on a negative delta plot slope of the Simon effect, Ridderinkhof (2002a, b) has developed a dual-route model which assumes the operation of an active inhibition mechanism that gradually overcomes an automatic route. Here I tested whether the global-to-local dynamics characterizes the composite face effect. I predict that a global-to-local dynamics should be manifested in a negative slope, entailing the reduction of the effect with time due to a coarse-to-fine representation (Sergent, 1986).

At this point it should be acknowledged that the application of delta plots is not without its critics. Zhang and Kornblum’s (1997) have argued that the delta plot “reflects the statistical properties of the pair of RT distributions and not necessarily functional hypotheses concerning mechanisms” (p. 155). They have shown that when the delta plot is linear, its slope is determined by the statistical relations between the variances of the two RT distributions. So, a positive slope emerges when the slower condition has higher variance than the faster condition, and a negative slope emerges when the opposite is the case. This criticism has been well taken in subsequent studies (Ridderinkhof, 2002a, b; Schwarz and Miller, 2012). One powerful approach to address this criticism has been to test hypotheses regarding processing mechanism using not one, but several converging measures (Ridderinkhof, 2002a, b). This is exactly what I have purported to do in the current study. In addition to the delta plots, I have also employed delta plots for error rates, which measure the error rates at different percentiles and plot them against the mean of the two RTs in those conditions (Ridderinkhof, 2002a). These type of delta plots are not vulnerable to Zhang and Kornblum’s criticism. Similarly, the CDFs and CAFs tools, which are not inflicted by this criticism, will serve as additional converging sources of evidence.^3^

### Linking Dynamic Aspects of Face Perception to Distributional Patterns

The goal of the current study is to utilize the distributional analyses to delineate the global-to-local and local-to-global hypotheses. The *global-to-local* (Sergent, 1986) and *local-to-global* hypotheses lead to distinct (and opposing) patterns of RTs and accuracy in the distributional analyses. As explained earlier, performance in the composite face task (and with face stimuli in general) is governed by two types of information – featural (local) and holistic (global). These two types of information are processed along two routes of information (Diamond and Carey, 1986; Sergent, 1986; Bartlett and Searcy, 1993; Rakover and Teucher, 1997; Macho and Leder, 1998; Rakover, 1998), and are available at different moments in time. Now, assume that a global route is processing information at an early stage, when the local route is still not active or is only weakly active. This entails that fast RTs or RTs in early percentiles will be affected by the operation of this global route and should therefore produce larger composite face effects. However, as the local route is becoming increasingly active, suppressing or replacing the information coming from the global route, its influence on performance will be more prominent. This will necessarily result in weaker composite face effects for longer RTs or later percentiles. The reduction in the composite face effect should be manifested in the distributional analyses. If we plot the size of the composite face effect as a function of time with RT or error rate as dependent variables, we would expect a negative slope. Note that a similar “activation-suppression” model has been described by Ridderinkhof (2002a, b) to account for the negative delta plots observed in the Simon task (De Jong et al., 1994). According to Ridderinkhof, performance in the Simon task is affected by two opposing routes. One is an automatic route that computes the (irrelevant) spatial location of the target, the other is a controlled route that computes the (relevant) color of the target (De Jong et al., 1994). The automatic route is very fast, operating at early processing stages, whereas the controlled route becomes increasingly effective at later processing stages, suppressing the activation of the automatic route. As a result, the Simon effect is strongest at early percentiles and gets progressively weaker for late percentiles. This account is very similar to the one proposed here. The global information is processed along fast automatic route, whereas the local information is processed along a slow controlled route. This asymmetry should produce a delta plot with negative slope.

The ‘local-to-global’ hypothesis leads to the exactly opposite predictions from the ‘global-to-local’ hypothesis. The featural (local) route is active at early stages, while the holistic (global) route is effective only at later stages. As a result, the size of the composite effect should be small to non-existent in the early percentiles (fast RTs) but increasingly larger in later percentiles (slower RTs), resulting in delta plots with positive slopes for RT and error rates. All these patterns should also be manifested in the CDFs as increasing or decreasing effect with time.

## Materials and Methods

### Participants

In the current study, 21 (20 female) undergraduate students were recruited from Ariel University’s pool of participants (mean age = 22.4, *SD* = 2.1). They received course credit for their participation. All participants reported normal or corrected-to-normal vision. All participants gave their written consent for participation.

### Stimuli

Stimuli were constructed from 40 faces published as part of Cornes et al. (2011). Faces were transformed into gray scale and were cut in half to produce 20 face tops and 20 face bottoms (see Figure 1). On every trial, the top and bottom parts of each face were randomly composed. A thin white line separated the face halves. Faces were presented as gray scale photos over black background. In the aligned faces, the two halves were presented exactly one above the other. In the misaligned faces, the top halve was shifted to the middle of bottom half (see Figure 1). Viewed at a fixed distance of 76 cm, the aligned images subtended 4.4° of visual angle, horizontally, and 2.3° vertically, whereas the misaligned images subtended 6.5° of visual angle, horizontally, and 2.3° vertically. To exclude the possibility that observers will use information from the different shapes of faces, all faces were presented within the same oval shape.

### Procedure and Design

Each trial started with a fixation cross that was presented for 500 ms at the center of the screen. The fixation was followed by a study face, which was presented for 200 ms. Rossion (2013) argued that if the exposure durations are long (several 100s of milliseconds, as in the current study) the composite face effect is likely to occur in the RT domain, while short presentation durations produce an effect only in the error rate. I have used this presentation duration because it is long enough to allow for effects in RTs, and because it is frequently used in other composite face studies. The study face was always an aligned composite face. The study face was then followed by a blank for 290 ms and then the test composite face appeared till response. No masking was used.

There were separate blocks for aligned and misaligned composite faces. These blocks alternated. Each block consisted of 80 trials in which all four possible combinations of study and test faces appeared. Thus, the relevant (top) halves and the irrelevant (bottom) halves of the study and test faces could create four possible type of trials: (a) top same + bottom same, (b) top different + bottom same, (c) top same + bottom different, and (d) top different + bottom different. These four types of trials were presented in equal frequency. Each block was repeated six times with the order of stimuli randomly decided by the computer. Overall each participant completed 960 experimental trials.

Participants were instructed to respond only to the top half of the test face and indicate by pressing one of two keys whether it is ‘same’ or ‘different’ from the study face’s top. Participants were asked to ignore the bottom half, which could also be ‘same’ or ‘different’ with the bottom half of the study face. The instructions highlighted both speed and accuracy. Participants were asked to press one key (“m”) if the top part was ‘same’ with the study face, and another key (“z”) if the top half was different from the study face. Response mapping was kept constant for all observers and across all conditions (aligned and misaligned).

In the data analyses, a congruent trial is a trial where top (relevant) and bottom (irrelevant) halves were both ‘same’ or both ‘different,’ whereas an incongruent trial is a trial where the top and bottom halves did not match, such that one half was ‘same’ and the other ‘different’ (see Richler and Gauthier, 2014).

## Results

### The Composite Face-Effect

**FIGURE 5:**
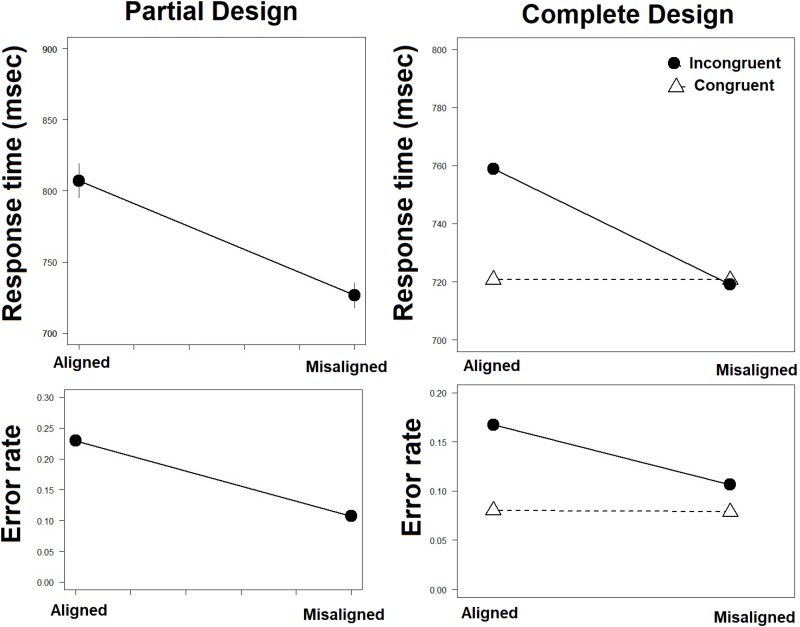
Mean RTs and Error Rates in the complete **(right)** and partial **(left)** designs.

Data appear in Supplementary Table S1. Data were censored such that RTs of 2.5 SD’s above the mean or RTs slower than 150 ms were removed from analysis. This procedure was held separately for each participant, for each experimental condition (Ulrich and Miller, 1994; Steinborn et al., 2017) to retain as many trials in the analysis. This resulted in the removal of 2.7% of the trials. Error trials (11%) were also removed in RT analyses. Mean RTs and Error rates are presented in Figure 5. The ANOVA with Congruity and Alignment as factors on mean RTs showed that the effect of alignment was significant [*F*(1,20) = 5.64, *MSE* = 8516,${\text{η}}_{\text{p}}^{2}$ = 0.22, *p* < 0.05], with faster RTs for misaligned than aligned faces. The congruency effect was significant [*F*(1,20) = 15.2, *MSE* = 11926,${\text{η}}_{\text{p}}^{2}$ = 0.43, *p* < 0.005], with faster RTs when both face halves were ‘same’ or both were ‘different.’ A significant Congruency × Alignment interaction [*F*(1,20) = 13.07, *MSE* = 9744,${\text{η}}_{\text{p}}^{2}$ = 0.36, *p* < 0.005] pointed to the modulation of this congruency effect by alignment. Further planned comparison showed that the interaction pattern was according to the predicted. A congruency effect was present in the aligned [*F*(1,20) = 14.46, *MSE* = 19873, ${\text{η}}_{\text{p}}^{2}$ = 0.39, *p* < 0.005], but not in the misaligned condition [*F* < 1]. These results replicate the standard complete design composite face effect (Richler and Gauthier, 2014) in the RT data.

A similar set of analyses was performed on mean error rates. The effect of alignment was significant [*F*(1,20) = 74.24, *MSE* = 0.068, ${\text{η}}_{\text{p}}^{2}$ = 0.97, *p* < 0.005], entailing more errors for aligned than misaligned faces. The congruency effect was significant too [*F*(1,20) = 9.70, *MSE* = 0.020, ${\text{η}}_{\text{p}}^{2}$ = 0.33, *p* < 0.01], pointing to lower error rate in the congruent than in the incongruent trials. This congruency effect was modulated by alignment, as indicated by a significant Congruency × Alignment interaction [*F*(1,20) = 21.85, *MSE* = 0.016, ${\text{η}}_{\text{p}}^{2}$ = 0.51, *p* < 0.0005]. Planned comparisons revealed that a congruency effect was present in the aligned condition [*F*(1,20) = 17.41, *MSE* = 0.036, ${\text{η}}_{\text{p}}^{2}$ = 0.42, *p* < 0.005], but not in the misaligned condition [*F* < 1]. These results demonstrate the presence of the complete design composite face effect also in the accuracy data.

A composite face effect in the partial design (Rossion, 2013) was computed as a difference in performance between aligned and misaligned conditions only for trials in which the top (relevant part) was ‘same’ and the bottom (irrelevant part) was ‘different.’ Figure 5 gives the mean RTs and mean error rate in the two conditions. The effect for RTs was significant [*t*(20) = 4.1, *p* < 0.005], with slower RTs in ‘same’ trials in the aligned compared to the misaligned condition. A comparable effect was found on error rates [*t*(20) = 9.16, *p* < 0.0005], such that more errors were committed in ‘same’ trials in the aligned than in the misaligned condition. These results confirm the presence of the composite face effect in the partial design (Rossion, 2013), in both RT and accuracy.

### Distributional Analyses

#### CDF and Delta Plots in the Complete Design

**FIGURE 6:**
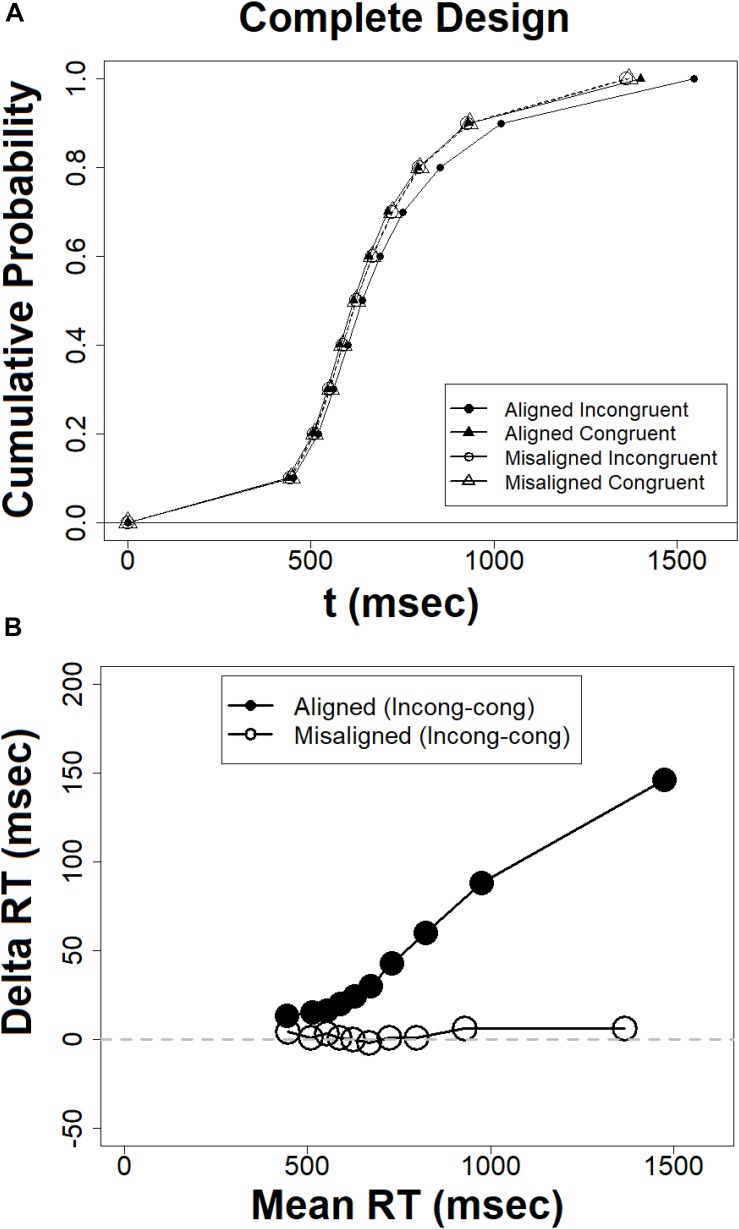
**(A)** Cumulative probability functions (CDFs) for the complete design, **(B)** delta plots for the complete design, the abscissa = (mean RT congruent + mean RT incongruent)/2.

The distributional analyses were based on computation of mean RT at a given percentile value using the R (R Core Team, 2013) function QUNATILE with the equally spaced 9 percentiles (0.1–0.9). This R function produces sample quantiles corresponding to the given probabilities. The smallest observation corresponds to a probability of 0 and the largest to a probability of 1. The CDFs and delta plots were derived and plotted for the complete design using R. First, note the CDFs plotted in Figure 6A. The CDFs for the aligned incongruent condition deviates significantly from the CDFs of the other three conditions, which are almost identical. The CDF for the aligned incongruent condition is shifted to the right, a pattern that reflects longer completion times and can readily explain the Congruency × Alignment interaction observed at the level of mean RTs (see Figure 5). Notably, the deviations of this CDF from the other three occurs at the later percentiles. We can use the CDFs to trace the temporal dynamics of the composite effect. The difference in cumulative probabilities between the aligned incongruent and congruent CDF in a given completion time (*t*) gives the size of the effect for that time. Viewed from this vantage point, the composite effect is minimal for short RTs but increases as RTs get slower. This pattern can result from effects of selective influence of the experimental manipulations on the skewness of the distributions. To assess selective influence on the later (and not the earlier) percentiles in the RT distribution, one should demonstrate that the effect exists in the *coefficient of variation of response times* (CVRT, Wagenmakers and Brown, 2007; cf. Steinborn et al., 2017, 2018). This measure reflects the skewness of the distribution as a ratio of the standard deviation to the mean (CVRT = SD/MRT × 100, see Flehmig et al., 2007). I computed the CVRTs values for each participant in each experimental condition and subjected them to ANOVA. Alignment exhibited a significant effect [*F*(1,20) = 5.36, *MSE* = 350, ${\text{η}}_{\text{p}}^{2}$ = 0.21, *p* < 0.05], with smaller CVRTs for misaligned than aligned faces. The Congruency × Alignment interaction [*F*(1,20) = 4.64, *MSE* = 60.7, ${\text{η}}_{\text{p}}^{2}$ = 0.18, *p* < 0.05] captured the modulation of congruency by alignment. Planned comparison revealed that a congruency effect with longer CVRTs in incongruent than in congruent trials, was present in the aligned [*F*(1,20) = 8.24, *MSE* = 98,${\text{η}}_{\text{p}}^{2}$ = 0.29, *p* < 0.01], but not in the misaligned condition [*F* < 1]. These results confirm the observations documented by the CDF, that the composite effect is selectively affecting the later percentiles.

This conclusion receives further support from the delta plot analyses, which are closely related to the CDFs in terms of their mathematical characteristics (Schwarz and Miller, 2012). Figure 6B presents the delta plots of the congruency effect in aligned and misaligned conditions. The delta function for the aligned faces is all positive and increasing, whereas the function for the misaligned faces remains roughly constant at zero. This visual impression has been corroborated by statistical analyses following the method used by De Jong et al. (1994) and Pratte et al. (2010). I first fit straight lines to individual’s delta plots, and then constructed contrasts across conditions on the resulting slope estimates. Delta plots slopes for aligned faces were significantly greater than zero [*t*(20) = 4.16, *p* < 0.001], whereas the slopes for the misaligned faces were not different from zero [*t*(20) = −0.75, *p* = 0.77]. It can also be noted that the differences between the two delta plots is zero at the fast RTs and increase gradually as RTs get slower, suggesting that the Alignment × Congruency interaction is increasing with time.

Taken together, both the CDFs, delta plot and CVRTs supported the same conclusion, namely that the composite face effect for RT in the complete design is very small or non-existent for very fast RTs but increasing as RTs get slower. This pattern is in sheer contrast to the global-to-local hypothesis (Sergent, 1986; Jacques and Rossion, 2009), and in agreement with the local-to-global hypothesis (Treisman and Gelade, 1980; Carbon and Leder, 2005).

#### CDF and Delta Plots in the Partial Design

**FIGURE 7:**
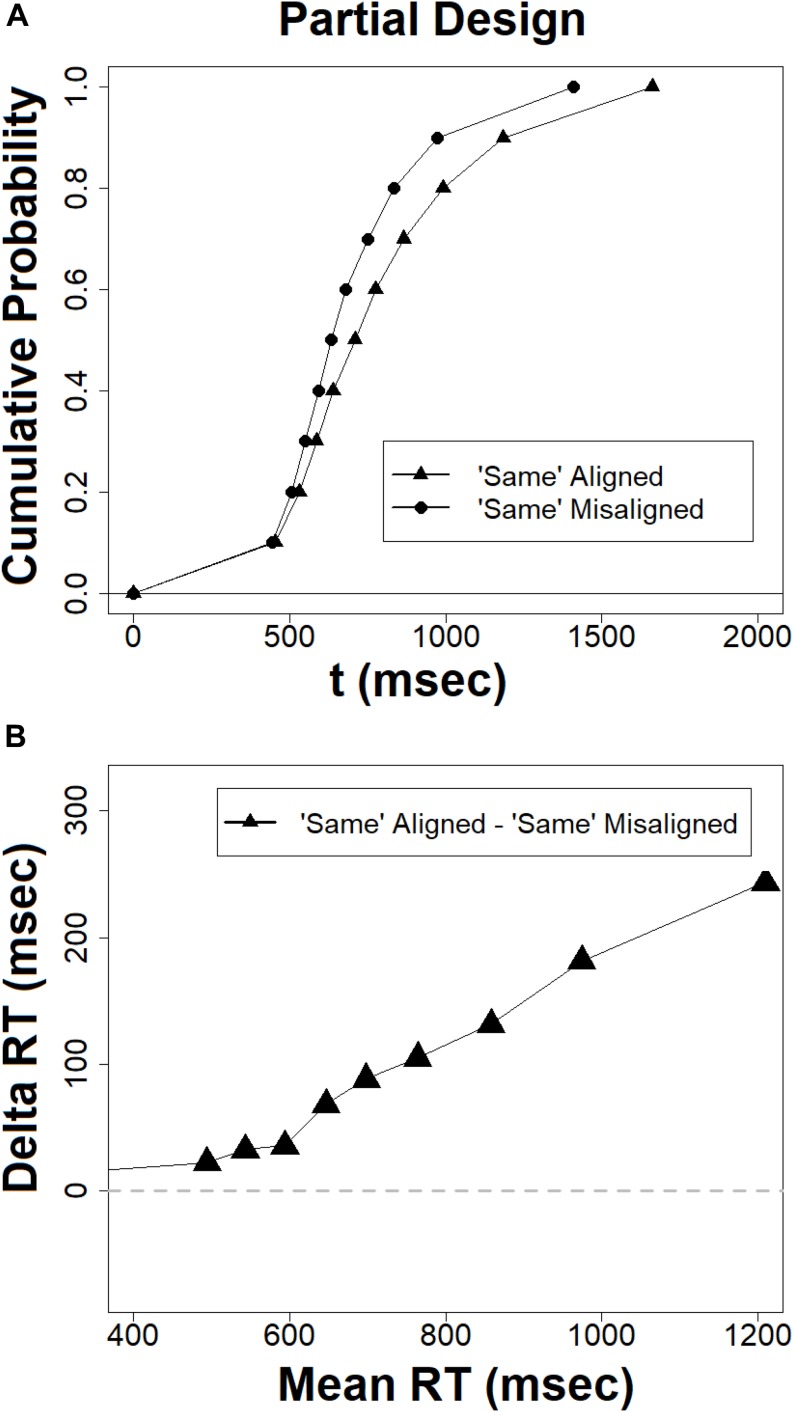
**(A)** Cumulative probability functions (CDFs) for the partial design, **(B)** delta plots for the partial design, the abscissa = (mean RT congruent + mean RT incongruent)/2.

The CDFs and delta plots were derived for the partial design measure which depicts the CDFs for trials in which the top is ‘same’ and the bottom is ‘different’ in aligned and misaligned conditions. As can be noted in Figure 7A, the ‘same’ misaligned CDF reaches its asymptote faster than the ‘same’ aligned CDF, an expected pattern given the finding of an RT composite face effect in the partial design (see Figure 5). The plot also uncovers the temporal dynamics of the composite effect in this measure. As can be noted, the effect is minimal to non-existent for fast RTs and then becomes increasingly larger as RTs get slower. To assess selective influence on the later percentiles in the RT distribution, one should demonstrate that the pertinent effect exists also in the *coefficient of variation of response times* (CVRT, cf. Steinborn et al., 2017, 2018). Comparing the CVRT values in the two critical conditions showed as expected larger values in the former than in the latter [*t*(20) = 1.94, *p* < 0.05]. These results confirm the observations documented by the CDF of selective influence of the composite effect on later percentiles.

This conclusion receives further support from the delta plot for RTs. Figure 7B presents the delta plot of the partial design composite effect. As can be noted, it is above zero and increasing with a positive slope. This observation was confirmed statistically by deriving the delta plot slopes for each participant and testing their deviance from zero [*t*(20) = 4.25, *p* < 0.005]. Both the CDFs and the delta plot for the partial design composite effect demonstrate that, in contrast to the global-to-local hypothesis (Hole, 1994; Meinhardt-Injac et al., 2010, 2011) and in line with the local-to-global hypothesis (Carbon and Leder, 2005) the allegedly holistic effect is minimal for fast RTs but gets progressively larger in slower RTs.

#### Conditional Accuracy Functions and Delta Plots for Error Rates in the Complete Design

**FIGURE 8:**
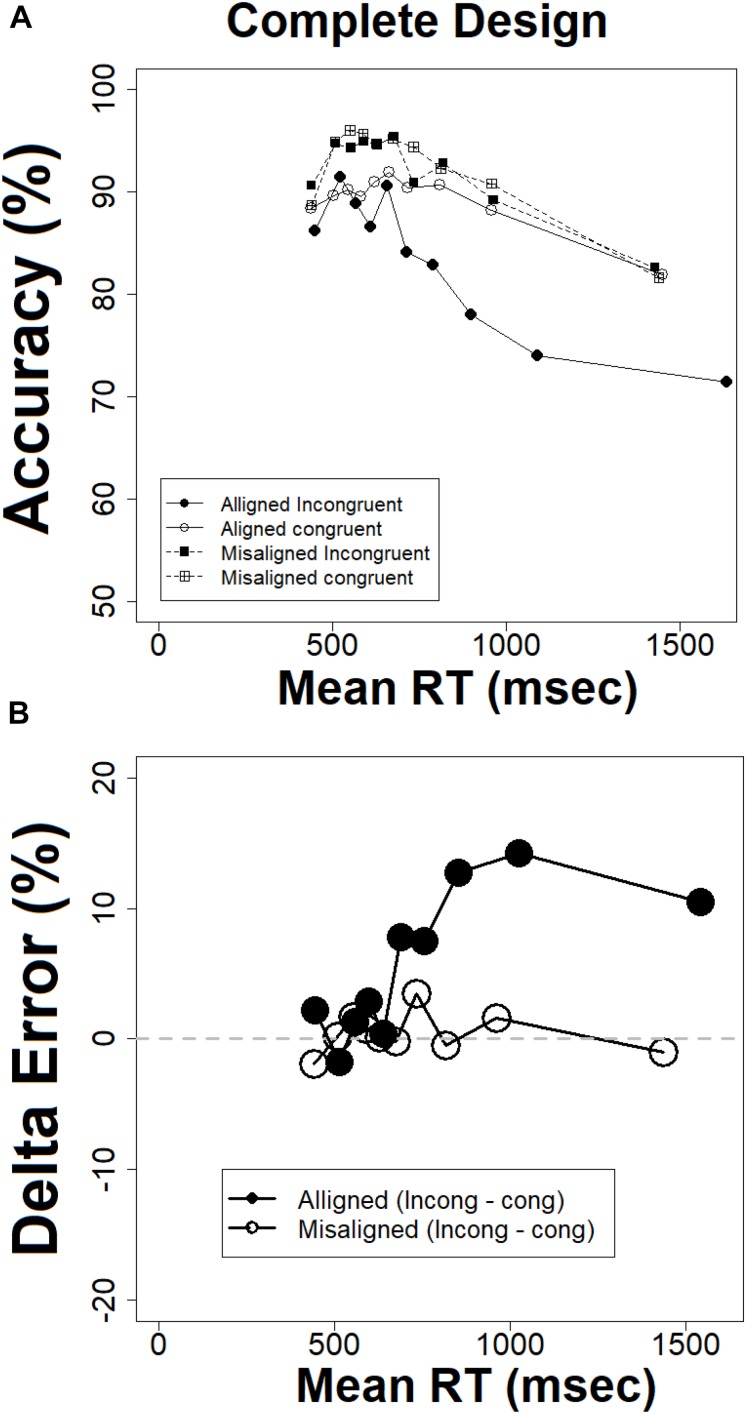
**(A)** Conditional accuracy functions for the complete design, **(B)** delta plots for error rates in the complete design, the abscissa = (mean RT congruent + mean RT incongruent)/2.

Conditional accuracy functions are depicted in Figure 8A. Most importantly, the composite effect for accuracy is absent for fast RTs but is increasingly growing as RTs get slower. This occurs, mainly due to the aligned incongruent condition which exhibits a strong tendency to deviate from the other three conditions. This is the exact pattern observed with the CDFs and delta plots for RTs. It is also interesting to note that the accuracy level in the four conditions is decreasing with time rather than increasing. This is an interesting finding as often accuracy improves with time. However, in the current matching task the accuracy of decisions on the target faces depended on successful retrieval of the study face from short term memory, which is subjected to decay with time (see also Walker-Smith, 1978). Moreover, this result also suggests the absence of a speed-accuracy tradeoff. Observers did not exchanged speed for accuracy. This can readily explain the overall decline in accuracy with time. Figure 8B depicts the delta plots for error, which describes how the composite face effect in error rates changes with speed of processing. As can be seen, these plots exhibit a positive slope for aligned composites which is increasing with time, whereas the error delta plot for the misaligned faces remains stable around zero. Statistical tests based on the same methods deployed earlier (Pratte et al., 2010) confirmed that the congruency effect in the aligned condition increases with time [*t*(20) = 3.93, *p* < 0.005]. In the misaligned condition the slope was also found to be increasing [*t*(20) = −2.30, *p* < 0.05]. But as can be noted in Figure 8B, the difference between the two slopes increases with time.

The results from the CDFs, the delta plots on RTs, conditional accuracy plots (CAFs), and delta plots for error converge on the same conclusion. The composite face effect in the “complete design” is minimal or non-existent for fast RTs but increases as RTs get slower. This conclusion is inconsistent with global-to-local hypothesis (Navon, 1977; Sergent, 1986; Schyns and Oliva, 1994; Sugase et al., 1999; Meinhardt-Injac et al., 2010, 2011), which predicted a decreasing composite face effect, with maximal effect for fastest RTs and minimal effect for slowest RTs. It is in line with a local-to-global account (Carbon and Leder, 2005).

#### Conditional Accuracy Functions and Error Delta Plots in the Partial Design

**FIGURE 9:**
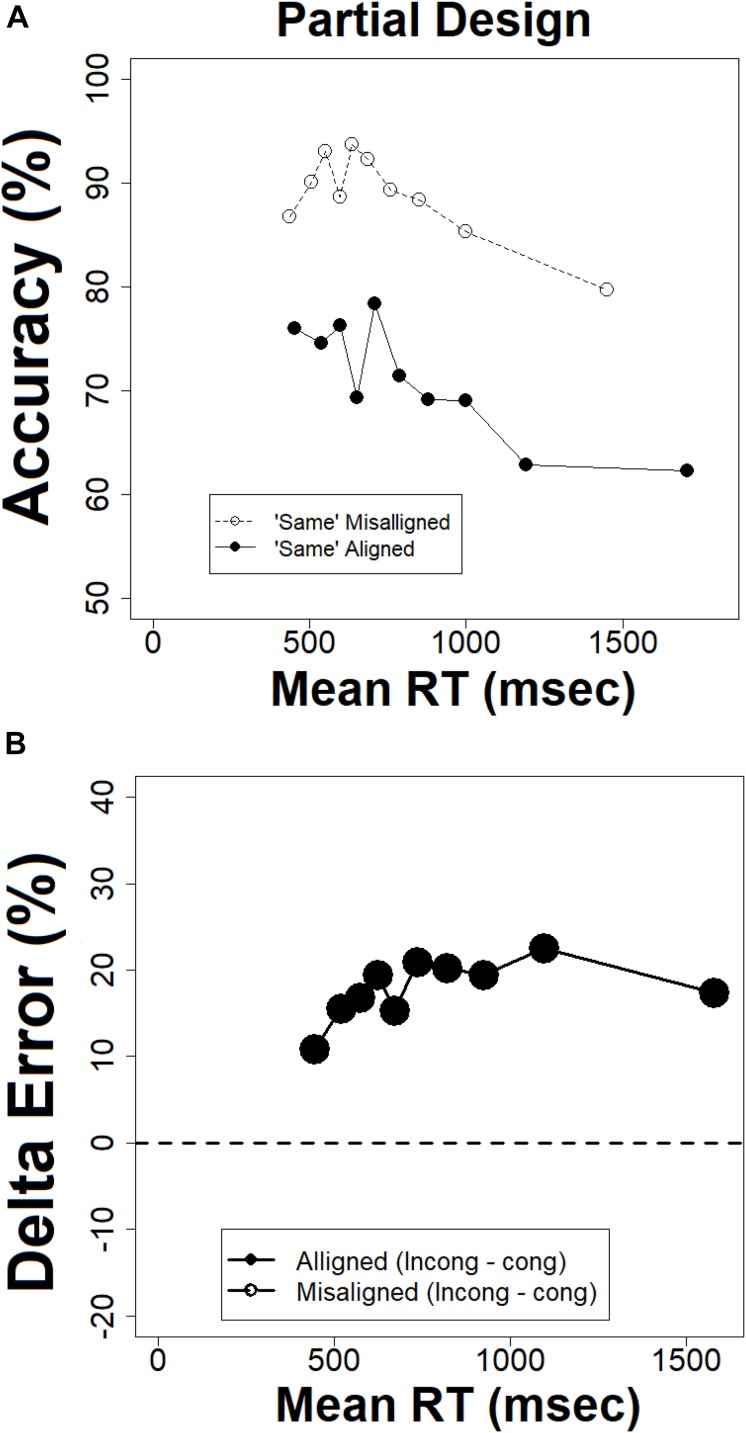
**(A)** Conditional accuracy functions in the partial design, **(B)** delta plots for error rates in the partial design, the abscissa = (mean RT congruent + mean RT incongruent)/2.

Figures 9A,B present the CAFs and delta plot for error respectively for the partial design. Most importantly, the size of the composite effect, as indicated by the difference between ‘same’ aligned and ‘same’ misaligned conditions is present in fast RTs and slightly increases as RTs get slower. This visual impression was further confirmed by the delta plot for error. Statistical analysis showed that the slope of the delta plot was positive [*t*(20) = 1.88, *p* < 0.05]. This pattern entails that the composite effect captured by error rates increases as responses get slower. The patterns documented by the CAFs and delta plot for error uncover a composite effect at the very fast RTs, while the CDF and delta plot for RTs do not. It seems that in the partial design the temporal dynamics of the composite effect is different when measured in RTs and accuracy. The RT pattern documents a complete absence of the effect at the outset, whereas the accuracy pattern shows that it exits in the very fast RTs. However, both measures are inconsistent with a global-to-local dynamics because in both cases the effect is not decreasing in magnitude with time.

In sum, the distributional analyses of the composite face effect in the complete (Richler and Gauthier, 2014) and partial (Rossion, 2013) designs showed that the composite face effect is small or non-existent in very fast responses but increases with time. This outcome bears important consequences for face perception theories. First, the current results provide strong evidence against the global-to-local hypothesis (Sergent, 1986; Goffaux and Rossion, 2006; Jacques et al., 2007; Jacques and Rossion, 2009; Meinhardt-Injac et al., 2010, 2011), according to which faces are represented initially a set of interdependent features or parts, and then progressively retain their independence. The current results support a local-to-global dynamics (Treisman and Gelade, 1980; Biederman, 1987; Macho and Leder, 1998; Carbon and Leder, 2005), according to which featural information is available early in processing whereas holistic or feature-conjunction information is available only later in processing. Second, the finding of a positive and increasing delta plots for the composite face effect offers tight constraints on future computational models of the effect (Schwarz and Miller, 2012). Third, the similarity between distributional characteristics of the complete and partial designs might suggest that they are not as remote and unrelated measures of the composite face illusion as has been argued (Rossion, 2013; Richler and Gauthier, 2014). The next section will further test this conjecture.

#### Relations Between Measures From the Complete and Partial Designs

Proponents of the complete (Richler and Gauthier, 2014) and partial (Rossion, 2013) have often argued that the two indices are utterly unrelated, to the extent they measure different things, and consequently lead to divergent conclusions. Richler and Gauthier (2014) reported very low and insignificant correlations between the two effects. But their conclusions rely on SDT variables, not the classic RTs and accuracy rates. Given the remarkable similarities in temporal dynamics between the effects, the question of their divergence begs a new look. Here, I have taken an individual differences approach (Rezlescu et al., 2017) and computed for each participant both the partial and complete effect size for both mean RT and mean error rate. The measure for the partial design was computed as a difference between aligned and misaligned trials in which the top was ‘same’ and the bottom ‘different’ (Rossion, 2013) according to the following:

RT⁢(Aligned)-RT⁢(Misaligned)

The complete design measure was computed as the mean interaction contrast of the Alignment × Congruency interaction according to the following:

=RT⁢(Aligned,Incongruent)-RT⁢(Aligned,Congruent)-[RT(Misaligned,Incongruent)-RT(Misaligned,Congruent)]

**FIGURE 10:**
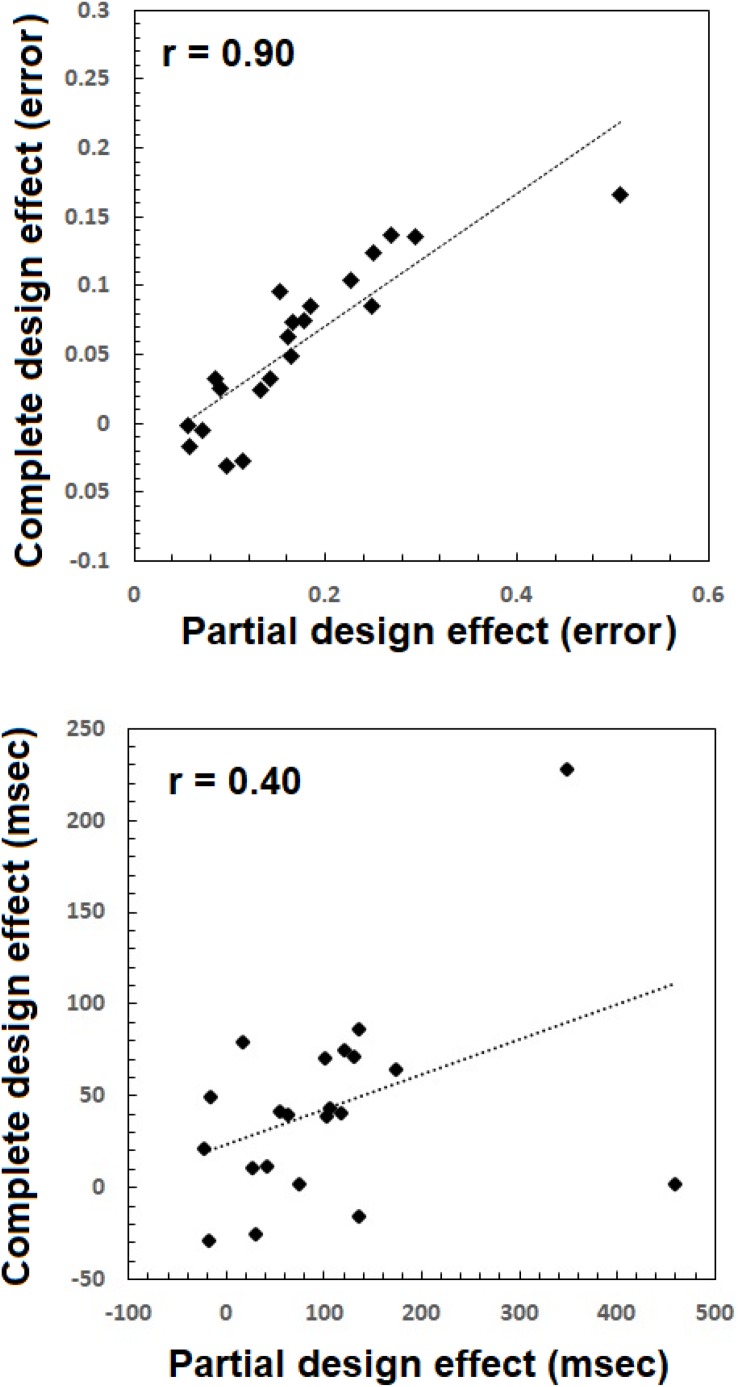
Composite face effects for each participant based on the partial design (abscissa) and the complete design (vertical axis) plotted one against the other, for error rate **(top)** and mean RTs **(bottom)**.

The computations for the mean error rates were similar, but with mean error rates instead of mean RT. The findings were surprising (see Figure 10). For accuracy the Pearson correlation was high, positive, and significant [*r* = 0.90, *t*(19) = 8.99, *p* < 0.005]. For RTs, the Pearson correlation was, medium, positive, and on the verge of significance [*r* = 0.40, *t*(19) = 1.93, *p* = 0.06]. These results are quite astounding considering the loud debate among proponents of the two measures. The current results demonstrate that the measures are not only exhibiting similar temporal dynamics but are correlated quite strongly.

## General Discussion

The current study offers what may be considered as the first fine-grained analysis of the RT distributions in the composite face illusion (Young et al., 1987). The distributional analyses included the CDF, CAFs, and delta plots for RTs and for error rates (Ridderinkhof, 2002a, b; Fitousi, 2015, 2016b). The work has been guided by two contrasting models of face perception, the *global-to-local* hypothesis (Navon, 1977; Sergent, 1986; Hole, 1994; Schyns and Oliva, 1994; Sugase et al., 1999; Meinhardt-Injac et al., 2010, 2011) and the *local-to-global* hypothesis (Tversky and Krantz, 1969; Diamond and Carey, 1986; Bartlett and Searcy, 1993; Rakover and Teucher, 1997; Macho and Leder, 1998; Rakover, 1998). These hypotheses postulate different time courses for the operation of configural and featural information, and as a result yield opposite predictions with respect to the temporal dynamics of the composite face effect. The distributional analyses converged on the same conclusion that the composite face effect is minimal or non-present at early stages of processing but in increasing in magnitude with time.

These results are in line with our new application of an earlier dual-route model (Ridderinkhof, 1997, 2002a,b) to the realm of face processing. The model consists of two routes: a local (analytic) information route which is fast and automatic and a global (holistic) information route which is slow and controlled. These two routes work in coalition to affect the observer’s decision in the composite face task. The local route is active at the early stages of processing and therefore participants are not affected by the irrelevant face half in fast RTs. However, as the global route becomes active, they become more and more prone to the influences of the irrelevant face half, hence the increase in the size of the composite effect as RTs get slower. The upshot is that the asymmetry in speed and function between the two routes results in early influence of the local (featural, part-based) information and a late influence of the global (holistic, configural) information. This model is inconsistent with the global-to-local hypothesis (Sergent, 1986), but is in full accordance with a local-to-global dynamics (Tversky and Krantz, 1969; Diamond and Carey, 1986; Bartlett and Searcy, 1993; Rakover and Teucher, 1997; Macho and Leder, 1998; Rakover, 1998). This model suggests that holistic face perception is not automatic but requires attention and resources to develop over time. This position is in line with attentional accounts of the composite face effect (Richler and Gauthier, 2014). It is also in accordance with non-holistic accounts which view the effect as an object-based effect (Fitousi, 2015, 2016b).

Rossion (2013) has argued that the complete design measure is not a face specific effect but rather a congruity effect akin to other congruity effects such as the Stroop (1935) and flanker (Eriksen and Eriksen, 1974) effects. While Richler and Gauthier argued that it is a true measure of holistic processing (Gauthier et al., 2018). They criticized the partial design measure which is based on only portion of the trials from the composite experiment. However, the current results reveal that both type of measures exhibit similar temporal dynamics, and more surprisingly are correlated to a large extent. Moreover, the underlying patterns support a local-to-global (feature-based) processing for both measures, which is more in line with the attentional account proposed by Richler and Gauthier (2014), than with the Gestalt view proposed by Rossion and colleagues (Jacques et al., 2007; Jacques and Rossion, 2009; Rossion, 2013) that assumes an initial strong Gestalt encoding of faces.

The finding of a positive increasing delta plots for both the complete and partial design measures can help us not only to compare between the two composite effects, but also to relate them to other attentional effects such as the Stroop and flanker, which have also been shown to exhibit positive and increasing delta plots (see Pratte et al., 2010). As stated earlier, the shape of delta plots can assist researchers in refuting or approving entire classes of process models (Schwarz and Miller, 2012). The fact that the composite face effect and the Stroop and flanker effects produce positive delta plots increases the likelihood that similar cognitive processes are involved.

How do the current results relate to previous studies? Meinhardt-Injac et al. (2010, 2011) have manipulated the exposure duration in the part-whole task. In their studies, global effects obtained for very brief exposure durations but not for long. Note however that beyond differences in method, these researchers used the part-whole task not the composite face task. A recent study by Rezlescu et al. (2017) found no correlation between performance scores in these tasks. But how can one explain the inconsistencies with previous composite face studies? Richler et al. (2009) found the effect to exist for very brief exposure durations and the effect remains stable with longer exposures. Hole (1994) found the composite effect for brief exposure duration (50 ms) but not for long (2 s) exposure duration. The differences in outcomes may be attributed to differences in methods. The exposure duration paradigm uses various exposure durations and thus can lead the observer to adopt different strategies in each duration. For example, observers in short duration may be faster but less accurate due to a more liberal setting of a decision boundary. The upshot is that exposure duration manipulations are not nearly optimal for studying the temporal dynamics because they confound the strategy by which observers perceive faces.

How do the current results inform theories of face perception? The finding of a local-to-global rather than a global-to-local processing dynamics counteracts claims raised by proponents of holistic processing (Goffaux et al., 2005; Goffaux and Rossion, 2006). The current results do not invalidate these claims, but they certainly cast doubts on the holistic mechanisms proposed (Sergent, 1986). Instead, the present outcome gives currency to alternative approaches such as the non-holistic processing of faces (Wenger and Ingvalson, 2002, 2003; Fitousi, 2015, 2016a; see also Cheng et al., 2018) or the feature-based approaches who postulate the independent processing of holistic and featural information (Tversky and Krantz, 1969; Bartlett and Searcy, 1993; Macho and Leder, 1998). According to the non-holistic approaches, the composite effect is comparable to other attentional effects such as the Stroop or flanker (Fitousi, 2015, 2016a). According to the latter, facial feature processing either runs in parallel to holistic information or precedes it. These approaches do not ascribe the holistic representation any precedence in time or in importance but assume equal weight of part-based and holistic information. If anything, the temporal dynamics uncovered here suggests that configural information might be perceived after the featural information is extracted.

Finally, the current work demonstrates how application of distributional analyses to a face recognition phenomenon can lead to interesting insights. Future work may apply these tools to other face perception phenomena, learning more about their commonalities and tying them to principled process models.

## Data Availability Statement

The datasets generated for this study are available on request to the corresponding author.

## Ethics Statement

The studies involving human participants were reviewed and approved by the Ariel University’s Ethics Committee. The patients/participants provided their written informed consent to participate in this study.

## Author Contributions

The author confirms being the sole contributor of this work and has approved it for publication.

## Conflict of Interest

The author declares that the research was conducted in the absence of any commercial or financial relationships that could be construed as a potential conflict of interest.

## Footnotes

1 The task deployed in SFT (Fitousi, 2015; Cheng et al., 2018) is slightly different from the traditional composite task as it requires observers to respond to both the top and bottom parts.

2 A reviewer noted that a standard Ratcliff diffusion model does not predict “true” experimental effects at late percentiles (which would correspond to a positively sloped delta plot) because the models produces a distribution that basically contains only scaling variability. Note that scaling variability refers to the proportion ratio of the individual standard deviation relative to the mean of an individual in a particular trial, measured by the coefficient of variation (see Wagenmakers and Brown, 2007). This means that one can only claim selective experimental effects on late percentiles, when it is shown that the coefficient of variation deviates from the proportion assumption (cf. Steinborn et al., 2017, 2018).

3 Another potential criticism is that the delta plot compares results of a process finished at earlier time with results from a process finished at later time. The delta plot can be closely approximated from its corresponding CDF by extracting the RTs for each percentile and then subtracting them and plotting them against their mean. The CDF can be viewed as the set of all differences between two cumulative probabilities of two processes at time *t*, but also as a set of all differences between two times (t_1_, t_2_) of the same cumulative probability.

## Supplementary Material

TABLE S1 The data of the experiment.
